# Molecular Insights Into Neutrophil Biology From the Zebrafish Perspective: Lessons From CD18 Deficiency

**DOI:** 10.3389/fimmu.2021.677994

**Published:** 2021-09-07

**Authors:** Almke Bader, Jincheng Gao, Thibaud Rivière, Bettina Schmid, Barbara Walzog, Daniela Maier-Begandt

**Affiliations:** ^1^Institute of Cardiovascular Physiology and Pathophysiology, Biomedical Center, Ludwig-Maximilians-Universität München, Planegg-Martinsried, Germany; ^2^Walter Brendel Center of Experimental Medicine, University Hospital, Ludwig-Maximilians-Universität München, Munich, Germany; ^3^Fish Core Unit, German Center for Neurodegenerative Diseases (DZNE), Munich, Germany

**Keywords:** zebrafish, neutrophils, integrins, inflammation, trafficking, CD18

## Abstract

Neutrophils are key players in innate immunity and originate from the bone marrow of the adult mammalian organism. In mammals, mature neutrophils are released from the bone marrow into the peripheral blood where they circulate until their recruitment to sites of inflammation in a multistep adhesion cascade. Here, adhesion molecules of the β_2_ integrin family (CD11/CD18) are critically required for the initial neutrophil adhesion to the inflamed endothelium and several post-adhesion steps allowing their extravasation into the inflamed tissue. Within the mammalian tissue, interstitial neutrophil migration can occur widely independent of β_2_ integrins. This is in sharp contrast to neutrophil recruitment in zebrafish larvae (*Danio rerio*) where neutrophils originate from the caudal hematopoietic tissue and mainly migrate interstitially to sites of lesion upon the early onset of inflammation. However, neutrophils extravasate from the circulation to the inflamed tissue in zebrafish larvae at later-time points. Although zebrafish larvae are a widely accepted model system to analyze neutrophil trafficking *in vivo*, the functional impact of β_2_ integrins for neutrophil trafficking during acute inflammation is completely unknown in this model. In this study, we generated zebrafish with a genetic deletion of CD18, the β subunit of β_2_ integrins, using CRISPR/Cas9 technology. Sequence alignments demonstrated a high similarity of the amino acid sequences between zebrafish and human CD18 especially in the functionally relevant I-like domain. In addition, the cytoplasmic domain of CD18 harbors two highly conserved NXXF motifs suggesting that zebrafish CD18 may share functional properties of human CD18. Accordingly, CD18 knock-out (KO) zebrafish larvae displayed the key symptoms of patients suffering from leukocyte adhesion deficiency (LAD) type I due to defects in *ITGB2*, the gene for CD18. Importantly, CD18 KO zebrafish larvae showed reduced neutrophil trafficking to sites of sterile inflammation despite the fact that an increased number of neutrophils was detectable in the circulation. By demonstrating the functional importance of CD18 for neutrophil trafficking in zebrafish larvae, our findings shed new light on neutrophil biology in vertebrates and introduce a new model organism for studying LAD type I.

## Introduction

Immune surveillance and host defense against invading pathogens are mediated by neutrophils patrolling within the mammalian circulation and the tissue (1, 2). Beyond their immediate immune function, neutrophils are involved in wound healing, metastasis, autoimmunity, and chronic inflammatory disease states (3). Neutrophil recruitment to sites of inflammation crucially depends on adhesion molecules of the β_2_ integrin family (CD11/CD18) that mediate adhesion, migration and extravasation as well as several host defense functions of these cells. β_2_ integrins are composed of the common β subunit CD18 and different α subunits namely CD11a, CD11b, CD11c, or CD11d (4). In humans, mutations in *ITGB2*, the gene for CD18, result in leukocyte adhesion deficiency (LAD) type I which is characterized by neutrophilia, impaired wound healing, and recurrent infections caused by compromised neutrophil recruitment to sites of infection (5). Mice lacking CD18 show similar defects (6–8).

In humans and mice, hematopoietic stem cells in the bone marrow give rise to granulocytic progenitor cells that further differentiate into neutrophils (9). Mature neutrophils are released into the circulation upon demand, with increasing amounts during inflammatory conditions (10–12). The lifespan of circulating neutrophils which is usually limited to hours or 1-2 days is tightly regulated by controlling neutrophil apoptosis and can be prolonged by e.g. cytokines and bacterial compounds (13, 14). Neutrophil trafficking to sites of inflammation depends on a concerted cascade of strictly regulated steps to recruit free-floating neutrophils from the blood stream into the inflamed tissue. The individual steps of neutrophil recruitment have been well-described in human and murine neutrophils and include neutrophil capture, rolling, slow rolling, arrest, adhesion strengthening, intraluminal crawling, transmigration as well abluminal crawling and interstitial migration (1). Neutrophil recruitment is initiated by capture and followed by subsequent rolling of neutrophils on the inflamed endothelial cells, a step mediated by selectins expressed on the endothelial cells and the P−selectin glycoprotein ligand-1 (PSGL-1) on neutrophils (1). Slow rolling depends on selectins as well as on lymphocyte function-associated antigen-1 (LFA-1, CD11a/CD18). Subsequent neutrophil arrest is induced by different chemokines including CXC motif chemokine ligand 8 (CXCL8) in humans and CXCL1 in mice, respectively, and mediated by binding of the β_2_ integrin LFA-1 to its ligands such as intercellular adhesion molecule 1 (ICAM-1) (1, 15). Outside-in signaling of β_2_ integrins enables the following steps of adhesion strengthening and cell spreading (1). Intravascular crawling to extravasation sites is mediated by macrophage-1 antigen (Mac-1, CD11b/CD18), followed by extravasation into the abluminal space where neutrophils display β_2_ integrin-dependent migration on activated pericytes until they eventually migrate interstitially to the site of inflammation (16, 17). In contrast to intraluminal and abluminal crawling and extravasation, subsequent interstitial migration within the tissue can occur independently of β_2_ integrins as shown in a mouse model depleted of integrins (18).

The zebrafish has emerged as a novel model to study neutrophil trafficking and function (19, 20). Zebrafish neutrophils execute the same host-defense functions such as phagocytosis, degranulation, or formation of neutrophil extracellular traps (NETs) as mammalian neutrophils (21–23). Moreover, zebrafish exhibit a range of advantages that make them an attractive model to study leukocyte trafficking *in vivo* (23). They are easy and relatively inexpensive to keep and give rise to a large number of offspring. Many genetic tools have successfully been established to generate mutant strains including gene editing using CRISPR/Cas9 (24, 25). Zebrafish larvae are transparent, allowing neutrophil visualization *in vivo* in intact organisms. Most importantly, the immune system of zebrafish and mammals is highly conserved (26, 27). While the innate immune system develops within the first 2 days post fertilization (dpf), the adaptive immune system only appears after 2 weeks, enabling the exclusive analysis of innate immune cells during this period in larval zebrafish (28). Disadvantages of this model include the partial lack of tools including antibodies which are still commercially unavailable for many proteins of interest. In addition, many mammalian genes have multiple orthologous genes in the zebrafish which may complicate genetic studies accordingly.

In zebrafish, first immune cell precursors are detected approximately 12 h post fertilization (hpf) during the primitive wave of hematopoiesis (23, 29). In the initial phase, the precursor cells give rise to macrophages and a further subset differentiates into neutrophils at approximately 33 hpf (30, 31). The definitive hematopoiesis starts at approximately 24 hpf when pluripotent precursors differentiate within the posterior blood island which expands to become the caudal hematopoietic tissue (CHT), the site functionally reminiscent of the fetal liver in mammals (30, 32, 33). The CHT gives rise to macrophages and neutrophils from 2 dpf onwards. From 4 dpf onwards, the kidney marrow begins to mature and becomes the site of definitive hematopoiesis in the adult zebrafish (34).

When neutrophils of zebrafish larvae leave the CHT, they predominantly migrate throughout the larvae in the interstitial space (33). In comparison to mammals, relatively few neutrophils are found in the circulation of zebrafish larvae whereas the majority of neutrophils is located within the tissue (35). The lifespan of neutrophils in zebrafish larvae was shown to be up to 5 days (36). In the first phase upon induction of inflammation by cutting or burning the tail fin, neutrophils mainly migrate within the tissue towards sterile injuries (37–39). Within 3-4 h post wounding, additional neutrophils are recruited from the circulation (40). The process of reverse migration of neutrophils from the inflamed tissue back into the vasculature has been discovered in zebrafish larvae and was subsequently reported in mice (41–43). Detailed information about the molecular players orchestrating neutrophil recruitment is still missing in zebrafish. P- and E-selectin expression have been described in zebrafish and it has been proposed that they have similar functions as their mammalian counterparts (44, 45). Furthermore, PSGL-1 is expressed in zebrafish and was shown to interact with human selectins (46). Similar to humans, CXCL8 binds to the CXC receptor 2 (CXCR2) in zebrafish directing neutrophils to sites of infection and mediating systemic recruitment of neutrophils from the CHT (47, 48). Previously, a zebrafish model of LAD type IV was generated by inducing a Rac2 mutation in zebrafish larvae (35). These Rac2 mutants displayed similar defects as found in human patients with the Rac2 mutation, including neutrophilia due to reduced neutrophil egress from the blood and reduced recruitment of neutrophils to sterile injury or bacterial infection. However, not all symptoms observed in human patients could be recapitulated in the zebrafish model (49).

To date, the putative role of β_2_ integrins for neutrophil trafficking in zebrafish is rather elusive. In this study, we generated a model for LAD type I in zebrafish by genome editing using CRISPR/Cas9 technology to shed light on the role of CD18 for neutrophil trafficking in this model. We used sequence alignments to demonstrate that the amino acid sequences of zebrafish and human CD18 share a high similarity, supporting the putative significance of the zebrafish as a model to study integrin biology. Accordingly, CD18 knock-out (KO) zebrafish larvae recapitulated the main symptoms observed in LAD type I patients including reduced neutrophil trafficking to sites of sterile inflammation despite the fact that an increased number of neutrophils was detectable in the circulation using spinning disk confocal microscopy. Thus, CD18 KO zebrafish larvae may represent an interesting model to study LAD type I in vertebrates.

## Materials and Methods

### Alignments

Sequence alignments of human, murine, and zebrafish CD18 and CD11b, using the longest isoforms from the UniProt database, were performed using Clustal Omega (50). Identity and similarity analyses were performed with the Sequence Manipulation Suite: Ident and Sim, using default settings (51).

### CRISPR/Cas9-Induced Generation of a CD18 Knock-Out Zebrafish Allele

Two different CD18 KO zebrafish lines were generated by CRISPR/Cas9-induced genome editing. To this end, two different guide RNAs (gRNAs) (gRNA1: CTGTGCATGGTGTAAAGAGT; gRNA2: TGCTGGTGGGAACACAGGGT) were designed using CHOPCHOP (52). gRNAs and Cas9 protein were ordered from Integrated DNA Technology, prepared according to the manufacturer’s protocol and injected into zebrafish embryos at the one cell stage. Zebrafish of the F_0_ generation were raised and outcrossed with Tg(*fli1:gfp*;*lyz:dsRed*) zebrafish. The F_1_ generation was fin-clipped and specific mutations were identified by sequencing. For the gRNA1, we selected a mutant allele with a deletion of 2 bp [Tg(*fli1:gfp*;*lyz:dsRed*;*itgb2^mde401^*), CD18 KO1], for the gRNA2 we selected a mutant allele with a deletion of 13 bp [Tg(*fli1:gfp*;*lyz:dsRed*;*itgb2^mde402^*), CD18 KO2] and bred them to homozygosity. DNA isolation was performed with the PCRBIO Rapid Extract PCR Kit (PCR Biosystems) according to the manufacturer’s instructions and PCR amplicons were sequenced to confirm the CD18 mutations.

### PCR

For PCR analysis, zebrafish larvae were euthanized by an overdose of tricaine (0.3 mg/ml, Pharmaq Ltd) and frozen at -80°C. RNA isolation was performed with the RNeasy Mini kit (Qiagen) and cDNA synthesis with the Maxima First Strand cDNA Synthesis Kit for RT-qPCR with dsDNase (Thermo Fisher Scientific) according to the manufacturers’ instructions. PCR was performed using the SsoAdvanced Universal SYBR Green Supermix (Bio-Rad Laboratories GmbH) and primer pairs against both WT and mutant CD18 for CD18 KO1 (forward 1: CACAGGTCTACACACAGGAGC, reverse 1: CTCCAGTCTTGGTGAAATTCAACT, product: 117 bp) and against WT CD18 only (forward 1: CACAGGTCTACACACAGGAGC, reverse 2: CTTGGTGAAATTCAACTCTTTAC, product: 110 bp) and primer pairs against both WT and mutant CD18 for CD18 KO2 (forward 3: AAACATCTCACAGCCGCCC, reverse 3: ACAGCAGCAAACTCTTCAGACA, product: 101 bp) and against WT CD18 only (forward 4: CGCCCATGACAGCTGTTTCT, reverse 4: GTGTAGACCTGTGTTCCCACC, product: 112 bp) in a peqSTAR PCR cycler (Peqlab/VWR). PCR products were separated in a 2% agarose gel and visualized with Midori Green (Nippon Genetics) in a gel documentation station (Peqlab).

### Zebrafish Strains

Zebrafish embryos of the transgenic lines Tg(*fli1:gfp*;*lyz:dsRed*), Tg(*fli1:gfp*;*lyz:dsRed*;*itgb2^mde401^*), and Tg(*fli1:gfp*;*lyz:dsRed*;*itgb2^mde402^*), referred to as CD18 wild-type (WT), CD18 KO1, and CD18 KO2, respectively, were kept in E3 medium (5 mM NaCl, 0.17 mM KCl, 0.33 mM CaCl_2_, 0.33 mM MgSO_4_, 0.00003% methylene blue) at 28.5 °C and were analyzed at 3 dpf and 5 dpf. 1-Phenyl 2-thiourea (0.003%, Sigma-Aldrich) was added to the medium 24 hpf. Raising and housing of adult zebrafish as well as the experiments described were performed in accordance with animal protection standards of the Ludwig-Maximilians-Universität München and approved by the government of Upper Bavaria (Regierung von Oberbayern).

### Microscopy

For counting of total neutrophil numbers and distribution, zebrafish larvae at 3 dpf and 5 dpf were euthanized by an overdose of tricaine (0.3 mg/ml) and fixed in 4% paraformaldehyde in PBS at 4 °C over night (53). Larvae were washed twice with PBS and imaged with an upright spinning disc confocal laser microscope (Examiner, Zeiss) equipped with a confocal scanner unit CSU-X1 (Yokogawa Electric Corporation), a CCD camera (Evolve, Photometrics) and a 5x/0.15NA objective (N-Achroplan, Zeiss) and Slidebook 6.0.13 Software (3i). Neutrophils were manually counted in a blinded fashion using the Cell Counter plugin in FIJI (54).

For live imaging of random neutrophil migration in the interstitial space as well as visualization of neutrophils in the circulation, zebrafish larvae were anaesthetized with 0.08 mg/ml tricaine (Pharmaq Ltd) in E3, mounted in 1.5% low melting agarose, and covered with 0.08 mg/ml tricaine in E3 for the duration of imaging (55). For neutrophil random migration, time series with an interval of 1 min and z-stacks with a step size of 10 µm were acquired for 15 min in the head region of zebrafish larvae 5 dpf with an upright spinning disc confocal laser microscope as described above and a 10x/0.3NA water immersion objective (N-Achroplan, Zeiss). 2D migration velocity and distances were calculated using FIJI with the Manual Tracking and the Chemotaxis Tool plugins (ibidi). For live imaging of neutrophils in the circulation after wounding, the tail fin was transected as described below for sterile wounding. Images were acquired 6 h post wounding. Time series of z-stacks with a step size of 5 µm were acquired for 5 min at the posterior caudal vein of zebrafish larvae at 3 dpf and 5 dpf as described before (48) with an upright spinning disc confocal laser microscope and a 10x/0.3NA water immersion objective as described above. The number of circulating neutrophils during the imaged time period was manually counted and calculated as circulating neutrophils/min. After imaging, zebrafish larvae were euthanized by an overdose of tricaine (0.3 mg/ml).

### Neutrophil Trafficking in Sterile Inflammation

Tail fin transection assays were performed with zebrafish larvae at 3 dpf or 5 dpf anaesthetized with 0.08 mg/ml tricaine. The tip of the tail fin was cut with a clean scalpel and the larvae were either fixed immediately with 4% paraformaldehyde in PBS (0 h) or placed back in E3 at 28.5 °C and anaesthetized and fixed 1, 3 or 6 h after wounding (56). Imaging was performed with a Leica M205 FA fluorescence stereo microscope, equipped with a DFC7000 T camera and a Planapo 1 x objective with the zoom set to 160 x. Neutrophil numbers within a 200 µm distance of the transected fin were manually counted using the Cell Counter plugin in FIJI.

## Results

### Integrin Alignments

The zebrafish genome harbors one orthologue (*itgb2*) of mammalian CD18. In addition to the *itgb2* gene, the *itgam* gene (CD11b) has been described in zebrafish, while orthologues for the *itgal* gene (CD11a), the *itgax* gene (CD11c) and the *itgad* gene (CD11d) do not exist in this model according to Ensembl (https://www.ensembl.org) and National Center for Biotechnology Information (NCBI) databases (https://www.ncbi.nlm.nih.gov). This fact implies that the important role of LFA-1 for neutrophil trafficking in the mammalian system may have no equivalent in zebrafish and that Mac-1 may represent the important β_2_ integrin in this model while LFA-1 is absent. To analyze this in more detail, we performed sequence alignments of the human, murine, and zebrafish amino acid sequences of CD18 (**Supplementary Figure 1**) and CD11b (**Supplementary Figure 2**). As expected, human and murine CD18 showed a high amino acid identity of 82.0% (**Table 1**). Zebrafish CD18 shared 49.8% identity and 64.4% similarity with human CD18. Since the extracellular I-like domain of mammalian CD18 is especially important for the regulation of ligand binding (57, 58) we aligned the I-like domains and found that this domain was not only highly conserved between human and mouse (94.6% identity and 96.2% similarity) but also between human and zebrafish (65.4% identity and 75.8% similarity), suggesting that zebrafish CD18 may function similar to human CD18. Strikingly, the two conserved NXXF motifs with critical importance for the function of the cytoplasmic domain of CD18 in the mammalian system are present in zebrafish as well (**Supplementary Figure 1**). Here, the conserved membrane-proximal NPLF domain of zebrafish CD18 was 100% identical to the mammalian system which is of eminent importance for CD18 function as it represents the binding site for the integrin regulator talin (59–61). Similarly, also the membrane-distal NXXF motif which represents the binding site for mammalian kindlin-3 was present in zebrafish (62). CD11b was less conserved between mammals and zebrafish. Here, human and murine CD11b were 74.6% identical and 84.1% similar, whereas zebrafish CD11b was only 25.8% identical and 36.1% similar to human CD11b. Again, the I domain of CD11b responsible for ligand binding was more conserved compared to the full length subunit with 78.8% identity and 84.4% similarity between human and mouse and 43.9% identity and even 58.3% similarity between human and zebrafish CD11b. Interestingly, a comparison between human CD11a and zebrafish CD11b revealed an identity of 21.7% and a similarity of 34.7% which was comparable to human and zebrafish CD11b alignments. These data suggest that zebrafish Mac-1 may share functional similarities to both LFA-1 and Mac-1 in mammals.

**Table 1:** Amino acid identity and similarity of murine and zebrafish CD18, CD11b, and CD11a in percent aligned to the respective human integrin (100%).

% to *Homo sapiens*	*Mus musculus*		*Danio rerio*	
	identity	similarity	identity	similarity
CD18	82.0	88.5	49.8	64.4
CD18 I-like domain	94.6	96.3	65.4	75.8
CD11b	74.6	84.1	25.8	36.1
CD11b I domain	78.8	84.4	43.9	58.3
CD11a	71.7	79.8	21.7^*^	34.7^*^
CD11a I domain	74.0	83.8	30.2^*^	53.1^*^

*Zebrafish CD11b was aligned to human CD11a.

### Generation of CD18 KO Zebrafish

**Figure 1:**
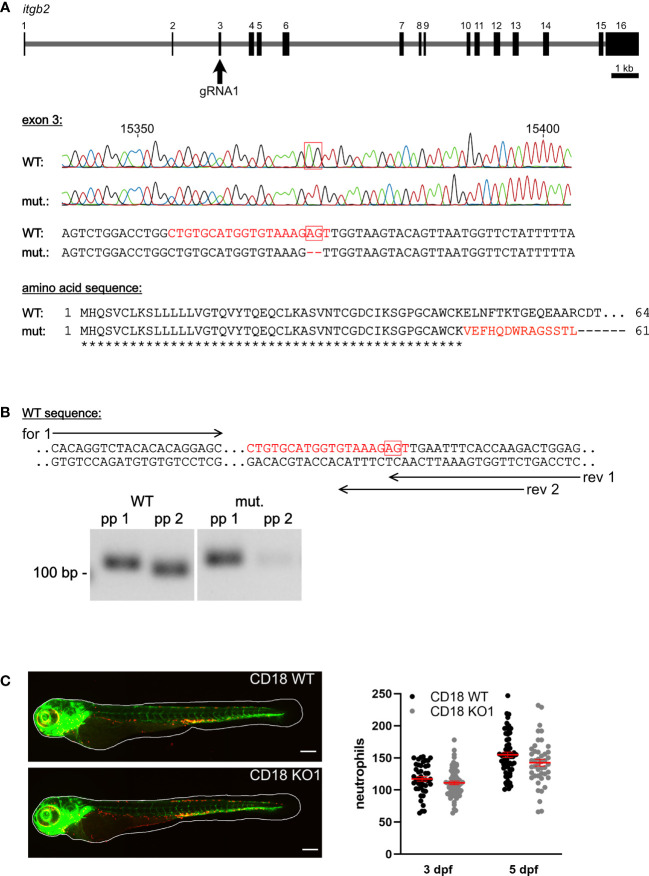
Generation of a CD18 KO zebrafish and analysis of neutrophil count. **(A)** Upper panel: Schematic of *itgb2* gene and target exon 3 of the gRNA1. Middle panel: Sequencing traces and partial genomic sequence of WT and CD18 mutant (mut.). Numbers indicate position within the gene. Red boxes show nucleotides deleted in the mutant. Highlighted is the target sequence of the gRNA in red. Lower panel: Predicted amino acid sequence of mutants aligned to WT sequence of the first 64 amino acids. Identical (*) and altered (red) amino acids are indicated. **(B)** Upper panel: Location of primer pairs for PCR analysis of WT and CD18 mutant mRNA expression analysis. Reverse (rev) primer 1 binds to WT and CD18 mutant sequence, reverse primer 2 only binds the WT sequence. Forward (for) primer 1 was combined with both reverse primers. Lower panel: Representative image of agarose gel electrophoresis of PCR products of WT and CD18 mutant cDNA with primer pairs (pp) 1 (forward primer 1, reverse primer 1) and 2 (forward primer 1, reverse primer 2), respectively, from zebrafish larvae at 5 dpf. Expected band sizes are 117 bp for pp 1 and 110 bp for pp 2. **(C)** Left: Exemplary maximum intensity projections of CD18 WT and CD18 KO1 zebrafish larvae at 3 dpf. Endothelial cells are shown in green, neutrophils in red. Scale bars represent 200 µm. Right: Total neutrophil counts in CD18 WT and CD18 KO1 zebrafish larvae at 3 dpf and 5 dpf. Mean ± sem of ≥ 44 individual larvae of ≥ 3 independent experiments. Unpaired t-test.

CD18 mRNA expression was detected in CD18 WT zebrafish larvae from 1-5 dpf using RT-PCR (data not shown). The CD18 KO1 and CD18 KO2 were generated using CRISPR/Cas9 genome editing in a transgenic zebrafish line expressing green fluorescence protein (GFP) under the *fli1* promoter and DsRed under the *lyz* promoter to specifically label endothelial cells and neutrophils, respectively (24, 25, 63, 64). gRNA1 (CD18 KO1) was chosen to target exon 3 of the zebrafish *itgb2* gene and a KO allele with a 2 bp deletion was selected which resulted in an early stop codon after amino acid 61 due to a frameshift mutation (**Figure 1A**). gRNA2 (CD18 KO2) was chosen to target exon 2 of the zebrafish *itgb2* gene and a KO allele with a 13 bp deletion was selected which resulted in an early stop codon after amino acid 43 (**Supplementary Figure 3A**). Reverse transcription PCR analysis using primer pairs located at the site of the 2 bp (**Figure 1B**) or 13 bp deletion (**Supplementary Figure 3B**) only binding to CD18 WT cDNA confirmed the absence of CD18 WT mRNA in the CD18 KO1 and CD18 KO2 zebrafish larvae, respectively. The CD18 KO1 and CD18 KO2 zebrafish were viable and fertile (data not shown).

### Neutrophils in CD18 KO Zebrafish Larvae at Steady State

**Figure 2:**
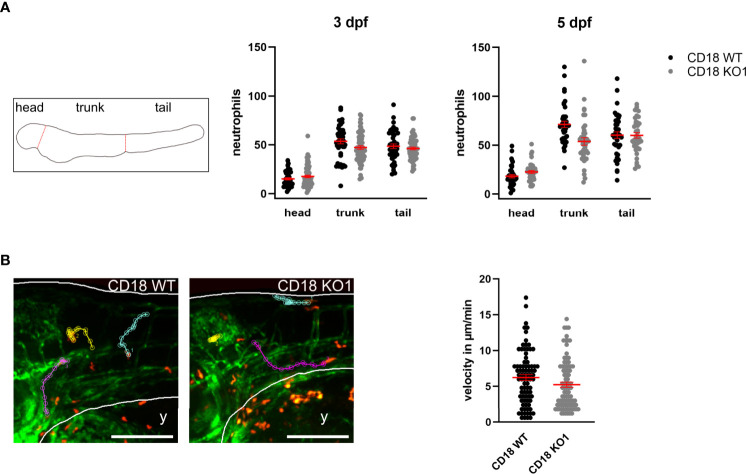
Analysis of neutrophil count and random migration at steady state. **(A)** Left: Schematic of zebrafish larvae for analyzing neutrophil distribution in head, trunk, and tail. Middle and right: Distribution of neutrophils in CD18 WT and CD18 KO1 zebrafish larvae at 3 dpf and 5 dpf. Mean ± sem of ≥ 44 individual larvae of ≥ 3 independent experiments. **(B)** Left: Exemplary maximum intensity projections of randomly migrating neutrophils in the head area of CD18 WT (left) and CD18 KO1 (right) zebrafish larvae at 5 dpf. Endothelial cells are shown in green. Neutrophils are shown in red. Y indicates yolk. Three representative migration tracks of neutrophils within 15 min are highlighted. Scale bars represent 200 µm. Right: Mean migration velocity of individual neutrophils. Mean ± sem of ≥ 84 individual neutrophils of ≥ 5 independent experiments. Unpaired t-test.

The CD18 KO larvae developed normally up to 5 dpf and did not show any obvious phenotypical differences compared to CD18 WT larvae. To study the consequences of CD18 deficiency on neutrophils in this model, we fixed CD18 WT and CD18 KO larvae at 3 dpf and 5 dpf and imaged them using confocal fluorescence microscopy. Neutrophils could clearly be observed as individual, DsRed labeled cells in CD18 KO larvae as expected (**Figure 1C**, **Supplementary Figure 3C**). Quantification of the total neutrophil number in the whole animal at 3 dpf and 5 dpf revealed no significant difference of neutrophil numbers in CD18 KO1 larvae compared to CD18 WT larvae. Next, we studied the overall distribution pattern of neutrophils within the zebrafish larvae in the head, trunk and tail region under normal conditions. Again, no striking differences were observed in neutrophil numbers in the different regions in the absence of CD18 in CD18 KO1 (**Figure 2A**). Similar results were obtained for CD18 KO2 (**Supplementary Figure 4A**). Random interstitial migration of neutrophils in non-injured zebrafish larvae was studied within the head region. Here, random migration velocity was similar in CD18 WT larvae (6.2 ± 0.4 µm/min) and CD18 KO1 larvae (5.2 ± 0.4 µm/min) at 5 dpf (**Figure 2B** and **Supplementary Video 1**). This was also true in CD18 KO2 larvae where no differences were observed in random migration velocity and Euclidian distance compared to CD18 WT larvae, suggesting that in accordance to the mammalian system CD18 was widely dispensable for interstitial migration in this model (**Supplementary Figure 4B**).

### Neutrophil Trafficking in CD18 KO Zebrafish Larvae During Acute Inflammation

**Figure 3:**
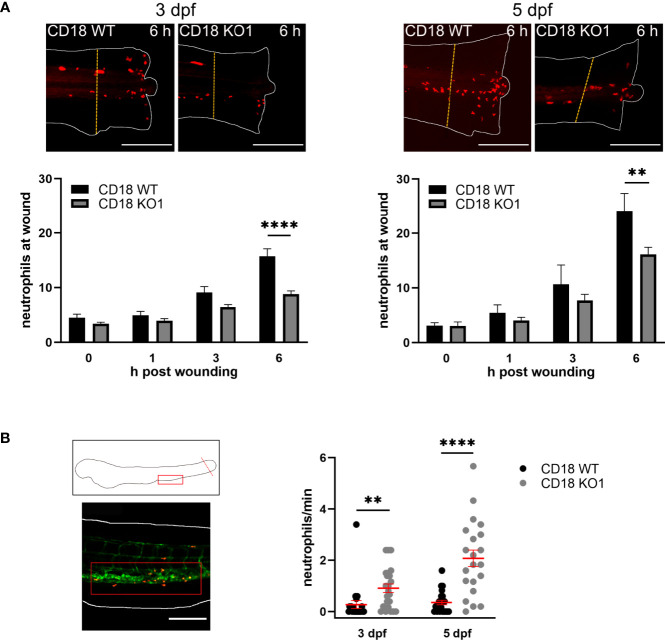
Neutrophil trafficking in CD18 KO zebrafish. **(A)** Upper panel: Exemplary maximum intensity projections of neutrophil recruitment 6 h after tail fin transection in CD18 WT and CD18 KO1 zebrafish larvae at 3 dpf (left) and 5 dpf (right). Neutrophils are depicted in red. The dashed lines indicate the 200 µm area in which the neutrophils were counted. Scale bars represent 200 µm. Lower panel: Quantification of neutrophil numbers at the transected tail fin in 3 dpf (left) and 5 dpf (right) CD18 WT and CD18 KO1 zebrafish larvae. Mean± sem of ≥ 8 individual larvae of ≥ 2 independent experiments. One-way ANOVA, *p* < 0.01: **, *p* < 0.0001: ****. **(B)** Schematic (upper left panel) and representative microscopic image (lower left panel) of the location of the posterior caudal vein (red box) that was imaged for quantification of circulating neutrophils. The dotted line indicates the tail fin transection. Scale bar represents 200 µm. Right panel: Number of neutrophils per min detected in the circulation 6 h after tail fin transection in CD18 WT and CD18 KO1 zebrafish larvae at 3 dpf and 5 dpf. Mean ± sem of ≥ 21 individual larvae of 3 independent experiments. Unpaired t-test, *p* < 0.01: **, *p* < 0.0001: ****.

To elucidate the functional impact of CD18 for neutrophil trafficking in inflamed zebrafish larvae, a tail fin transection assay to analyze neutrophil recruitment upon sterile wounding was performed. The tip of the tail fin was cut in zebrafish larvae at 3 dpf and 5 dpf and the number of neutrophils recruited to the wound was quantified at 0, 1, 3, and 6 h after the onset of the experiment. No differences were observed after 1 h and 3 h (**Figure 3A**). However, significantly less neutrophils were recruited to the tail fin after 6 h in CD18 KO1 larvae as compared to CD18 WT larvae at 3 dpf (8.8. ± 0.6 versus 15.7 ± 1.4) and 5 dpf (16.1 ± 1.3 versus 24.1 ± 3.3) pointing towards a neutrophil recruitment defect upon sterile injury in zebrafish larvae at this time point. Similar results showing a significant reduction of neutrophil accumulation at sites of lesion were obtained for CD18 KO2 zebrafish larvae when compared to CD18 WT larvae 6 h post wounding (**Supplementary Figure 5A**). This effect that CD18 was only critically required at later time points after the onset of inflammation may be due to the fact that neutrophil accumulation up to 3-4 h post wounding predominantly occurs by interstitial migration whereas neutrophil recruitment after that time point is enabled by extravasation from blood vessels (40). Thus, our data suggest that recruitment of neutrophils from the circulation may involve CD18 in zebrafish larvae. Interestingly, the number of neutrophils detectable in the circulation using spinning disk confocal microscopy was significantly increased in CD18 KO1 larvae 6 h post wounding compared to CD18 WT larvae at 3 dpf and 5 dpf (**Figure 3B** and **Supplementary Video 2**). Similar results were obtained in CD18 KO2 (**Supplementary Figure 5B**). Thus, the defective accumulation of neutrophils at sites of lesion despite an increased number of neutrophils in the circulation may reflect the impaired extravasation efficiency of CD18 deficient neutrophils which is a hallmark of LAD type I in patients and CD18 deficiency in the murine system (5, 8).

## Discussion

Taken together, we generated CD18 KO zebrafish lines to study the role of β_2_ integrins for neutrophil trafficking in the zebrafish model. CD18 deficiency in humans leads to LAD type I which is characterized by neutrophilia and recurrent infections due to a recruitment defect of neutrophils to sites of inflammation (5). To prove our hypothesis that zebrafish and human CD18 may have similar functions, we performed sequence alignments between human, murine, and zebrafish CD18 and CD11b. These alignments indicated that CD18 was highly conserved between species. In line with these findings, CD18 of human, murine and origin zebrafish share a common ancestor and is found within the same cluster of the phylogenetic tree (65). However, CD11b seems to be less conserved between zebrafish and mammals. In our alignments, both human CD11a and CD11b showed similar identities with zebrafish CD11b, suggesting that zebrafish CD11b may compensate for the absence of CD11a and thereby it may at least partially fulfill the role of CD11a in mammals. In mice, LFA-1 and Mac-1 play distinct roles for neutrophil adhesion and migration. Both LFA-1 and Mac-1 contribute to slow rolling of neutrophils on the endothelium but LFA-1 is predominantly important for neutrophil arrest (66–68). Studies with KO mice further revealed that LFA-1 deficient mice displayed leukocytosis and impaired neutrophil adhesion and extravasation, similar to CD18 deficient animals (69). However, Mac-1 also contributes to neutrophil adhesion and migration but it cannot fully compensate for the loss of LFA-1. Mammalian Mac-1 has important roles in mediating neutrophil defense functions such as phagocytosis and degranulation and promotes neutrophil apoptosis and thereby plays an important role in different inflammatory diseases such as glomerulonephritis, or thrombohemorrhagic vasculopathy (70–72). In addition, Mac-1 attenuates FcγRIIA-dependent neutrophil recruitment in response to deposited immune complexes which is key to organ damage in a model of systemic lupus erythematosus and thus prevents excessive inflammation, demonstrating that Mac-1 has diverse and partially opposing roles in mammals (73, 74). Thus, we speculate that the important roles of LFA-1 and Mac-1 in mice may be accomplished at least to some extend by only Mac-1 in zebrafish.

Our zebrafish model presented with an increased number of neutrophils detectable in the circulation by spinning disk confocal microscopy which is in line with Rac2 deficient zebrafish larvae as a model for LAD type IV which also show increased neutrophil numbers in the circulation (35). CD18 deficient humans and mice display increased neutrophil numbers in the bone marrow and in the circulation (5, 8, 75). The neutrophilia in CD18 KO mice is not mainly caused by enhanced neutrophil survival or the inability of neutrophils to leave the vascular compartment. Here, the genetic absence of CD18 causes the disruption of a feedback loop involving G-CSF and IL-17 and drives granulopoiesis as neutrophils cannot migrate into peripheral tissues (76). In addition to an increased release of neutrophils from the bone marrow into the circulation, reduced extravasation of neutrophils at sites of inflammation and delayed apoptosis of neutrophils may contribute to neutrophilia upon CD18 deficiency in mammals to some extend (77). In zebrafish, evidence has been reported that a CD18 knock-down may induce the expansion of hematopoietic pluripotent stem cells in the CHT which opens the possibility that CD18 may influence granulopoiesis in this model as well (78).

Analysis of random interstitial migration of neutrophils in the head region demonstrated that CD18 was widely dispensable for migration in this model. This is in line with findings in mice where integrins are not required for interstitial leukocyte migration (18). In sharp contrast, we did observe a strong and significantly reduced neutrophil recruitment to sites of sterile injury at 6 h post wounding in the CD18 KO zebrafish larvae. As neutrophil migration was largely unaffected by CD18 deficiency, a cell-intrinsic impairment of the migratory capacity can be excluded as relevant mechanism for defective neutrophil recruitment to the site of the tail fin injury 6 h after the onset of inflammation. At this time point, neutrophils are mainly recruited from the circulation (40). Thus, the impaired neutrophil accumulation in the inflamed tissue despite elevated neutrophil numbers in the circulation suggests that neutrophils may not be able to egress from the blood stream in the genetic absence of CD18. However, the exact molecular mechanisms underlying this extravasation defect in zebrafish larvae requires further investigation. This is especially true for the endothelial counterreceptors of the β_2_ integrins which are largely unknown in this model (79). In accordance with our findings, small molecules specifically interfering with Mac-1 have been reported to reduce the recruitment of neutrophils in wounded tail fins in zebrafish larvae within 4 h after the onset of the experiment further supporting of role for Mac-1 in neutrophil extravasation (80).

Our zebrafish CD18 KO model presents a global KO of the *itgb2* gene similar to the patients’ situation and therefore effects of the deletion of CD18 in other tissue than neutrophils cannot be ruled out. However, *itgb2* is expressed in zebrafish larvae only in leukocytes as previously published by Xue et al. (78). Here, *itgb2* expression is restricted to the region of the CHT and hematopoietic cells at 2, 3, and 4 dpf as shown with whole-mount *in situ* hybridization (WISH). Furthermore, expression of *itgb2* is restricted to neutrophils, macrophages, and thymus according to the UCSC cell browser database (81). Since we analyzed zebrafish larvae at 3 dpf and 5 dpf no cells of the adaptive immune system are present which start to develop two weeks after fertilization.

Of note, zebrafish larvae represent an embryonic model and the data obtained in the present study are therefore not directly transferable to the situation in human patients and murine models. Hence, future studies characterizing the CD18 KO zebrafish lines at the adult stage can shed additional light on the role of CD18 on neutrophil trafficking in zebrafish. Only recently, the existence of a substantial pool of neutrophils present in the tissue under steady state conditions has been reported in mammals which modulate organ function but can also influence pathological states including e.g. tumor cell infiltration (2). Beyond the immediate role of neutrophils in innate immunity, our knowledge of the homeostatic function of tissue neutrophils only starts to emerge and zebrafish larvae may represent an extremely valuable tool to decipher the versatile roles of neutrophils under steady state conditions by live imaging in the intact organism.

## Data Availability Statement

The original contributions presented in the study are included in the article/**Supplementary Material**. Further inquiries can be directed to the corresponding authors.

## Ethics Statement

The animal study was reviewed and approved by Regierung von Oberbayern Maximilianstraße 39 80538 München.

## Author Contributions

AB carried out research, analyzed data and wrote the manuscript. JG and TR carried out research and analyzed data. BS participated in generation of the zebrafish knock-out. BW analyzed data and wrote the manuscript. DM-B designed the study, carried out research, analyzed data and wrote the manuscript. All authors contributed to the article and approved the submitted version.

## Funding

This work was supported by grants from the Deutsche Forschungsgemeinschaft [SFB 914/A02 (DM-B and BW) and Z03 (BW)].

## Conflict of Interest

The authors declare that the research was conducted in the absence of any commercial or financial relationships that could be construed as a potential conflict of interest.

## Publisher’s Note

All claims expressed in this article are solely those of the authors and do not necessarily represent those of their affiliated organizations, or those of the publisher, the editors and the reviewers. Any product that may be evaluated in this article, or claim that may be made by its manufacturer, is not guaranteed or endorsed by the publisher.
