# Supplementary material for: Molecular Insights Into Neutrophil Biology From the Zebrafish Perspective: Lessons From CD18 Deficiency
Source: Front Immunol. 2021 Sep 7;12:677994. doi: 10.3389/fimmu.2021.677994 (PMC8453019; doi:10.3389/fimmu.2021.677994)
Supplement: Supplementary file 1 [file DataSheet_1.pdf]

frontiers

# *Supplementary Material*

**Molecular Insights Into Neutrophil Biology From the Zebrafish Perspective: Lessons From CD18 Deficiency**


**Almke Bader[1,2†], Jincheng Gao[1,2†], Thibaud Rivière[1,2], Bettina Schmid[3], Barbara Walzog[1,2*], Daniela Maier-Begandt[1,2*]**

**[1]Institute of Cardiovascular Physiology and Pathophysiology, Biomedical Center, Ludwig-Maximilians-Universität München, Planegg-Martinsried, Germany**

**[2]Walter Brendel Center of Experimental Medicine, University Hospital, Ludwig-Maximilians-Universität München, Munich, Germany**

**[3]Fish Core Unit, German Center for Neurodegenerative Diseases (DZNE), Munich, Germany**

[*]Correspondence: Daniela Maier-Begandt, Daniela.maier@lrz.uni-muenchen.de
Barbara Walzog, Walzog@lrz.uni-muenchen.de

[†]These authors have contributed equally to this work

# Supplementary Figures

## Supplementary Figure 1

### CD18

```
H. sapiens           MLGLRPPL-LALVGLLSLG--CVLSQECTKFKVSSCRECIESGPGCTWCQKLNFTGPGDPDSIRCDTRPQLLMRGCAADDIMDPTSLAET     87
M. musculus          MLGLRPSLLLALAGLFFLG--SAVSQECTKYKVSSCRDCIQSGPGCSWCQKLNFTGPGEPDSLRCDTRAQLLLKGCPADDIMDPRSIANP     88
D. rerio             ---MHQSVCLKSLLLLLVGTQVYTQEQCLKASVNTCGDCIKSGPGCAWCKELNFTKTGEQEAARCDTVKLLLDRGCSKENIINPQNIFQN     87
                        ::  : *    *: :*     .::* * .*.:* :**:*****:**::****  *: :: ****    ** :**   ::*::* .: :

H. sapiens           QED----HNGGQKQLSPQKVTLYLRPGQAAAFNVTFRRAKGYPIDLYYLMDLSYSMLDDLRNVKKLGGDLLRALNEITESGRIGFGSFVD     173
M. musculus          EFD----QRGQRKQLSPQKVTLYLRPGQAAAFNVTFRRAKGYPIDLYYLMDLSYSMLDDLNNVKKLGGDLLQALNEITESGRIGFGSFVD     174
D. rerio             VVNKALVAGKDPVQIQPQEIQLNLRPGMPQTFQLRFKRAEGYPVDLYYLMDLSYSMKDDLANVKNLGNDILEKLNRITGNARIGFGSFVD     177
                       :           *:.**:: * ****   :*:: *:**:***:************ ***  ***:**.*:*. **.**  ..*********

H. sapiens           KTVLPFVNTHPDKLRNPCPNKEKECQPPFAFRHVLKLTNNSNQFQTEVGKQLISGNLDAPEGGLDAMMQVAACPEEIGWRNVTRLLVFAT     263
M. musculus          KTVLPFVNTHPEKLRNPCPNKEKACQPPFAFRHVLKLTDNSNQFQTEVGKQLISGNLDAPEGGLDAIMQVAACPEEIGWRNVTRLLVFAT     264
D. rerio             KTLLPYTDTNEAKLKHPCSDKNEPCQPAFGFQHVLPLTANGNDFKELVKQQHISGNLDPPEGSLDAIMQAAVCLKEIGWGNSTRLLVLAT     267
                     **:**:.:*:   **::** :*:: *** *.*:*** ** *.*:*:   * :* ****** ***.***:**.*.* :**** * *****:**

H. sapiens           DDGFHFAGDGKLGAILTPNDGRCHLEDNLYKRSNEFDYPSVGQLAHKLAENNIQPIFAVTSRMVKTYEKLTEIIPKSAVGELSEDSSNVV     353
M. musculus          DDGFHFAGDGKLGAILTPNDGRCHLEDNMYKRSNEFDYPSVGQLAHKLSESNIQPIFAVTKKMVKTYEKLTEIIPKSAVGELSDDSSNVV     354
D. rerio             DDGFHMAGDGKLAAILEPNKETCQLDKNKYSKSNIWDYPSVGQVARKLEEQNIQPIFAVTKKMETVYTELSKLIPKSAVGVLSEDSNNVV     357
                     *****:******.*** **.  *:*:.* *.:** :*******:*:** *.*********.:*  ..* :*:::******* **:**.***

H. sapiens           QLIKNAYNKLSSRVFLDHNALPDTLKVTYDSFCSNGVTHRNQPRGDCDGVQINVPITFQVKVTATECIQEQSFVIRALGFTDIVTVQVLP     443
M. musculus          QLIKNAYYKLSSRVFLDHSTLPDTLKVTYDSFCSNGASSIGKSRGDCDGVQINNPVTFQVKVMASECIQEQSFVIRALGFTDTVTVQVRP     444
D. rerio             NLIVEAYNNLTSEVIMAHDALPNFISVKYSSKCTGGKTFS--DNGKCDNVNIGLEVIFDVTVTVDRCINDQSFSIGPLGFNEKLKVNVQT     445
                     :** :** :*:*.*:: *.:**: :.*.*.* *:.* :     .*.**.*:*.  : *:*.* . .**::*** *  ***.: :.*:*

H. sapiens           QCECRCRDQSRDRSLCHGKGFLECGICRCDTGYIGKNCECQTQGRSSQELEGSCRKDNNSIICSGLGDCVCGQCLCHTSDVPGKLIYGQY     533
M. musculus          QCECQCRDQSREQSLCGGKGVMECGICRCESGYIGKNCECQTQGRSSQELERNCRKDNSSIVCSGLGDCICGQCVCHTSDVPNKEIFGQY     534
D. rerio             LCECNCGDPPGVHPHCKGQGKVVCGSCKCNDGFIGQLCECTIGKKDEASFKAQCRKDNG-TECEGKGDCVCGRCQCHLTEGN-GHFYGEH     533
                      ***.* *    :  * *:* : ** *:*: *:**: ***    :.. .:: .*****.   *.* ***:**:* ** ::     ::*::

H. sapiens           CECDTINCERYNGQVCGGPGRGLCFCGKCRCHPGFEGSACQCERTTEGCLNPRRVECSGRGRCRCNVCECHSGYQLPLCQECPGCPSPCG     623
M. musculus          CECDNVNCERYNSQVCGGSDRGSCNCGKCSCKPGYEGSACQCQRSTTGCLNARLVECSGRGHCQCNRCICDEGYQPPMCEDCPSCGSHCR     624
D. rerio             CECDDEHCEKFDNKQCAG--HGKCKCGKCECDDGYEGTACHCAKSDNECKIDS-HVCYNRGKCVCNQCECERGYKGPYCKTCPTCQRPCQ     620
                     ****  :**:::.: *.*  :* * **** *. *:**:**:* ::   *        * .**:* ** * *. **: * *: ** *   *

H. sapiens           K-YISCAECLKFEKGPFGKNCSAACPGLQLSNN-PVKGRTCKERDSEGCWVAYTLEQQDGMDRYLIYVDESRECVAGPNIAAIVGGTVAG     711
M. musculus          DNHTSCAECLKFDKGPFEKNCSVQCAGMTLQTI-PLKKKPCKERDSEGCWITYTLQQKDGRNIYNIHVEDSLECVKGPNVAAIVGGTVVG     713
D. rerio             Q-SGSCVECFAFQSGPFEKNCSLACKHLTIKIVDKLVKSDCRVKDKEGCWMAFTMTEELGFDLYSVSVLKDRECPEGPNIIAIVGGSLAG     709
                     .   **.**: *:.*** ****  *  : :.    :    *: :*.****:::*: :: * : * : * .. **  ***: *****::.*

H. sapiens           IVLIGILLLVIWKALIHLSDLREYRRFEKEKLKSQWNNDNPLFKSATTTVMNPKFAES-     769
M. musculus          VVLIGVLLLVIWKALTHLTDLREYRRFEKEKLKSQWNNDNPLFKSATTTVMNPKFAES-     771
D. rerio             VALIGLLMLLIIKGVLYASDLREWRRFEKDRKHEKTSGTNPLFQNATTTVQNPTFSGDS     768
                     :.***:*:*:* *.: : :****:*****:: :.: .. ****:.***** **.*: .
```

**Supplementary Figure 1: Sequence alignment of the human, murine, and zebrafish integrin CD18.** The highly conserved I-like domains are shaded in gray. The transmembrane domain is shown in blue. The highly conserved NXXF motifs are highlighted in yellow and green, respectively. Identical (*), strongly similar (:) and weakly similar (.) amino acid residues are indicated below the alignment.

## Supplementary Figure 2

### CD11b

```
H. sapiens      -MAL----RVLLLTALTLCHGFNLDTENAMTF----QENARGFGQSVVQLQGSRVVVGAPQEIVAANQRGSLYQCDYSTGSCEPIRLQVP    81
M. musculus     -MTL----KALLVTALALCHGFNLDTEHPMTF----QENAKGFGQNVVQLGGTSVVVAAPQEAKAVNQTGALYQCDYSTSRCHPIPLQVP    81
D. rerio        MMDWYSYISFCIFCVSQSVMAFNIDPASWKTFTGPSQSQNVAFGYKVIQKDTSSLIVSDPLIQINQDKRGQIYNCEVAKGTCTQLNITVP    90
                 *         :. .     .**:*     **    *.:  .** .*:*   : ::*. *      :: * :*:*: :.. *  : : **

H. sapiens      VEAVNMSLGLSLAATTSPPQLLACGPTVHQTCSENTYVKGLCFLFGSNLRQQPQKFPEALRGCPQEDSDIAFLIDGSGSIIPHDFRRMKE    171
M. musculus     PEAVNMSLGLSLAVSTVPQQLLACGPTVHQNCKENTYVNGLCYLFGSNLLRPPQQFPEALRECPQQESDIVFLIDGSGSINNIDFQKMKE    171
D. rerio        SEAVNMSLGFSMVQHPQSSKLAICGPTIPKNCTTATNYRGMCFIGNSGDFGPPIPK-SKYRDCL-GQIDIAFLLDGSGSIGIYDFTVMKG    178
                 ********:*:.      :*  ****: :.*.  *  .*:*:: .*.     *     .  * *   : **.**:******   **  **

H. sapiens      FVSTVMEQLKKSKTLFSLMQYSEEFRIHFTFKEFQNNPNP-RSLVKPITQLLGRTHTATGIRKVVRELFNITNGARKNAFKILVVITDGE    260
M. musculus     FVSTVMEQFKKSKTLFSLMQYSDEFRIHFTFNDFKRNPSP-RSHVSPIKQLNGRTKTASGIRKVVRELFHKTNGARENAAKILVVITDGE    260
D. rerio        FVTNVIRRFIERDAQFAIAQYSNDCDIHYNFNDLKLDDGTWESKVANIPYHEGGTFTASAIQKLVNYLFTPNGGTRPSAKKILVVITDGE    268
                **:.*:.:: : .: *:: ***::  **:.*:::: : .   .* *  *    * * **:.*:*:*. **  ..*:* .* **********

H. sapiens      KFGDPLGYEDVIPEADREGVIRYVIGVGDAFRSEKSRQELNTIASKPPRDHVFQVNNFEALKTIQNQLREKIFAIEGTQTGSSSSFEHEM    350
M. musculus     KFGDPLDYKDVIPEADRAGVIRYVIGVGNAFNKPQSRRELDTIASKPAGEHVFQVDNFEALNTIQNQLQEKIFAIEGTQTGSTSSFEHEM    350
D. rerio        SHDRNL-LKDAASQAEKNSIVRFAIGVGKAFDYYNAREELNTIASDPDTDYVFKVTDFNALKNILQKLEGNIIAIEGTQTSGDSS-RMEF    356
                ...  *  :*.  :*:: .::*:.****.**   ::*.**:****.*  ::**:* :*:**:.* ::*. :*:*******.. ** . *:

H. sapiens      SQEGFSAAITSNGPL-LSTVGSYDWAGGVFLYTSKEKSTFINMTRVDSDMNDAYLGYAAAIILRNRVQSLVLGAPRYQHIGLVAMFRQNT    439
M. musculus     SQEGFSASITSNGPL-LGSVGSFDWAGGAFLYTSKDKVTFINTTRVDSDMNDAYLGYASAVILRNRVQSLVLGAPRYQHIGLVVMFRENF    439
D. rerio        AQDGFSTAFTSSGSVLVSAVGAFQWKGGYRDNSQEG--SF-----QTGSEHESYQGYSMAVATVSLTSFAILGAPRYQHRGQVTISRIGT    439
                :*:***:::**.* : :.:**:::* **    :.:   :*        .. :::* **: *:   . ..  :******** * *.: * .

H. sapiens      GMWESNANVKGTQIGAYFGASLCSVDVDSNGSTDLVLIGAPHYYEQTRGGQVSVCPLPRGRARWQCDAVLYGEQGQPWGRFGAALTVLGD    529
M. musculus     GTWEPHTSIKGSQIGSYFGASLCSVDMDADGNTNLILIGAPHYYEKTRGGQVSVCPLPRGRARWQCEALLHGDQGHPWGRFGAALTVLGD    529
D. rerio        NEVQPLD-SPMPQIGSYFGAEVCVVDLNSDSYTDLLLVSAPTYTESEREGKVFVYSFTSWSRLFFSDVVLVGMAG-QRGRFGSSLASPAD    527
                .  :         ***:****.:* **::::. *:*:*:.** * *. * *:* *  :       : .:.:* *  *   ****::*:  .*

H. sapiens      VNGDKLTDVAIGAPGEEDNRGAVYLFHGTSGSGISPSHSQRIAGSKLSPRLQYFGQSLSGG-QDLTMDGLVDLTVGAQGHVLLLRSQPVL    618
M. musculus     VNGDKLTDVAIGAPGEQENQGAVYIFYGASIASLSASHSHRIIGAHFSPGLQYFGQSLSGG-KDLTMDGLMDLAVGAQGHLLLLRAQPVL    618
D. rerio        LNGDGYRDVLVGAPLEEEGQGSIYIFNGRDGG-IISSYSQRIAGSSVKSGLQYFGVSLSQSSPDQTADSLPDIAAGSKGAVLLLRSRPIM    616
                :***   ** :*** *::.:*::*:* * . . :  *:*:** *: ..  ***** *** .  * * *.* *::.*::* :****::*::

H. sapiens      RVKAIMEFNPREVARNVFECNDQVVKGKEAGEVRVCLHVQKSTRDRLREGQIQSVVTYDLALDSGRPHSRAVFNETKNSTRRQTQVLGLT    708
M. musculus     RLEATMEFSPKKVARSVFACQEQVLKNKDAGEVRVCLRVRKNTKDRLREGDIQSTVTYDLALDPVRSRIRAFFDETKNNTRRRTQVFGLM    708
D. rerio        LLETMVSYTPSKISTIQQDCLIPLQ-----TTLTVCFTMKGYR---HHRRGLNAKIAYNITLDAKRQAYRAFFSAKERL-LSDVIDIGST    697
                 ::: :.:.* :::     *   :        : **: ::       :.  ::: ::*:::**  *   **.*. .:.      .  :*

H. sapiens      QTCETLKLQLPNCIEDPVSPIVLRLNFSLVGTPLSAFGNLRPVLAEDAQRLFTALF---PFEKNCGNDNICQDDLSITFSFMSLDCLVVG    795
M. musculus     QKCETLKLILPDCVDDSVSPIILRLNYTLVGEPLRSFGNLRPVLAMDAQRFFTAMF---PFEKNCGNDSICQDDLSITMSAMGLDTLVVG    795
D. rerio        EACNNHNFSIEACPEDALNPLSNQLKFTFEGLPSSQMQNLRPILLPEIKTTSDHDVRVHLCKVTSGNDALLT----VGYITVGF------    777
                : *:. :: :  * :* :.*:  :*:::: * *   : ****:*  : :      .      : ..*** :      :    :.:

H. sapiens      GPREFNVTVTVRNDGEDSYRTQVTFFFPLDLSYRKVSTLQNQRSQRSWRLA-CESASSTEVSGALKSTSCSINHPIFPENSEVTFNITFD    884
M. musculus     GPQDFNMSVTLRNDGEDSYGTQVTVYYPSGLSYRKDSASQNPLTKKPWFVKPAESSSSSEGHGALKSTTWNINHPIFPANSEVTFNVTFD    885
D. rerio        -------------------------------TYY---------------------------------------------------------    780
                                               :*

H. sapiens      VDSKASLGNKLLLKANVTSENNMPRTNKTEFQLELPVKYAVYMVVTSHGVSTKYLNFTASENTSRVMQHQYQVSNLGQRSLPISLVFLVP    974
M. musculus     VDSHASFGNKLLLKAIVASENNMSRTHKTKFQLELPVKYAIYMIVTSDESSIRYLNFTASEMTSKVIQHQYQFNNLGQRSLPVSVVFWIP    975
D. rerio        ------------------------------------------------------------------------------------------    780

H. sapiens      VRLNQTVIWDRPQVTFSENLSSTCHTKERLPSHSDFLAELRKAPVVNCSIAVCQRIQCDIPFFGIQEEFNATLKGNLSFDWYIKTSHNHL    1064
M. musculus     VQINNVTVWDHPQVIFSQNLSSACHTEQKSPPHSNFRDQLERTPVLNCSVAVCKRIQCDLPSFNTQEIFNVTLKGNLSFDWYIKTSHGHL    1065
D. rerio        ------------------------------------------------------------------------------------------    780

H. sapiens      LIVSTAEILFNDSVFTLLPGQGAFVRSQTETKVEPFEVPNPLPLIVGSSVGGLLLLALITAALYKLGFFKRQYKDMMSEGGPPGAEPQ      1152
M. musculus     LLVSSTEILFNDSAFALLPGQESYVRSKTETKVEPYEVHNPVPLIVGSSIGGLVLLALITAGLYKLGFFKRQYKDMMNEAAPQDAPPQ      1153
D. rerio        ----------------------------------------------------------------------------------------      780
```

**Supplementary Figure 2: Sequence alignment of the human, murine, and zebrafish integrin CD11b.** The I domains are shaded in gray. Identical (*), strongly similar (:) and weakly similar (.) amino acid residues are indicated below the alignment.

## Supplementary Figure 3

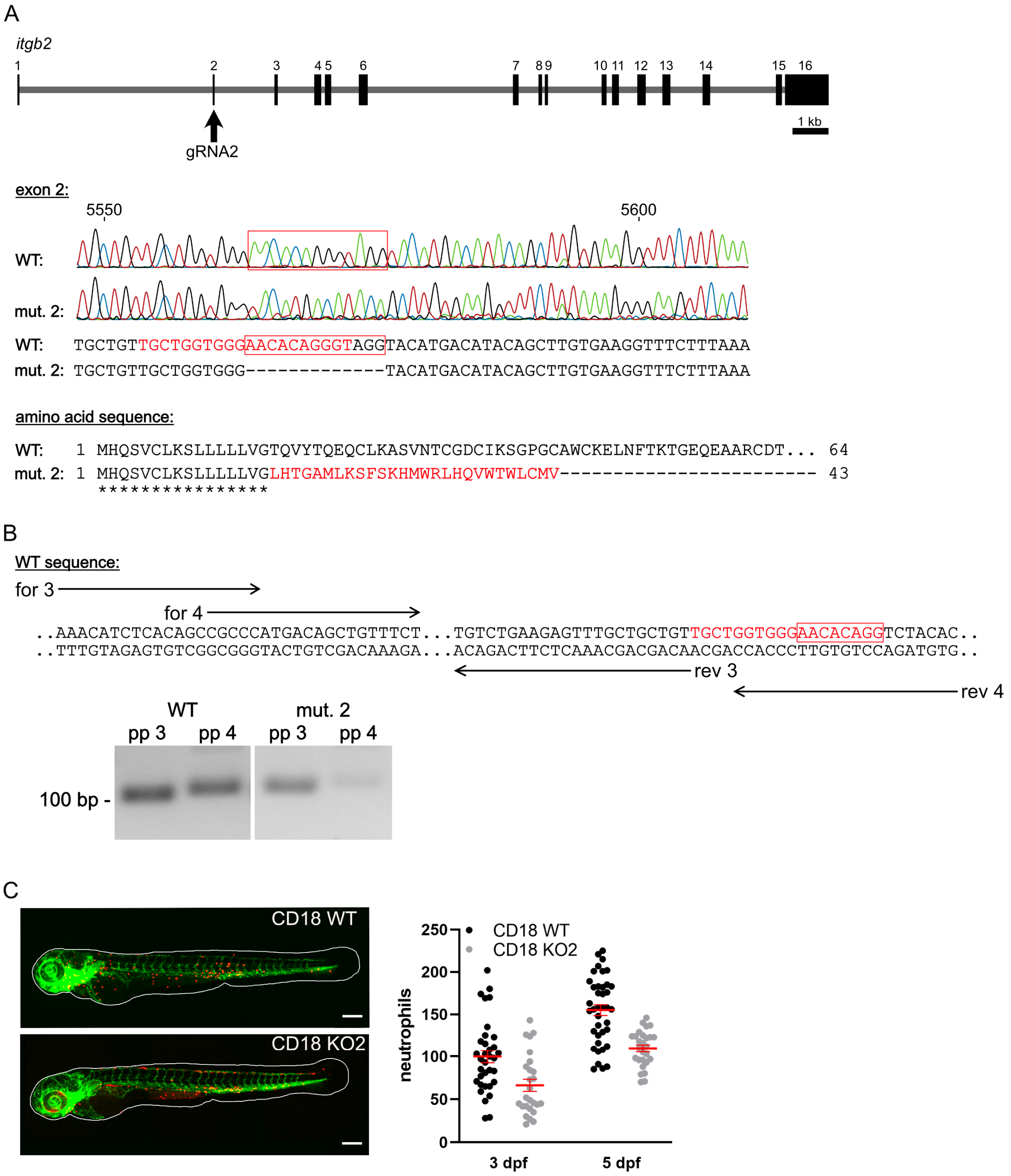


**Supplementary Figure 3: Generation of CD18 KO2 zebrafish.** (**A**) Upper panel: Schematic of *itgb2* gene and target exon 2 of the gRNA2. Middle panel: Sequencing traces and partial genomic sequence of WT and CD18 mutant 2 (mut. 2). Numbers indicate position within the gene. Red boxes show nucleotides deleted in the mutant. Highlighted is the target sequence of the gRNA in red. Lower panel: Predicted amino acid sequence of mutants aligned to WT sequence of the first 64 amino acids. Identical (*) and altered (red) amino acids are indicated. (**B**) Upper panel: Location of primer pairs for PCR

analysis of WT and CD18 mutant mRNA expression analysis. Reverse (rev) primer 3 binds to WT and CD18 mutant sequence, reverse primer 4 only binds the WT sequence. Lower panel: Representative image of agarose gel electrophoresis of PCR products of WT and CD18 mutant cDNA with primer pairs (pp) 3 (forward primer 3, reverse primer 3) and 4 (forward primer 4, reverse primer 4), respectively, from zebrafish larvae at 5 dpf. Expected band sizes are 101 bp for pp 3 and 112 bp for pp 4. (**C**) Left: Exemplary maximum intensity projections of CD18 WT and CD18 KO2 zebrafish larvae at 3 dpf. Endothelial cells are shown in green, neutrophils in red. Scale bars represent 200 µm. Right: Total neutrophil counts in CD18 WT and CD18 KO2 zebrafish larvae at 3 dpf and 5 dpf. Mean ± sem of ≥ 26 individual larvae of ≥ 3 independent experiments.

**Supplementary Figure 4**

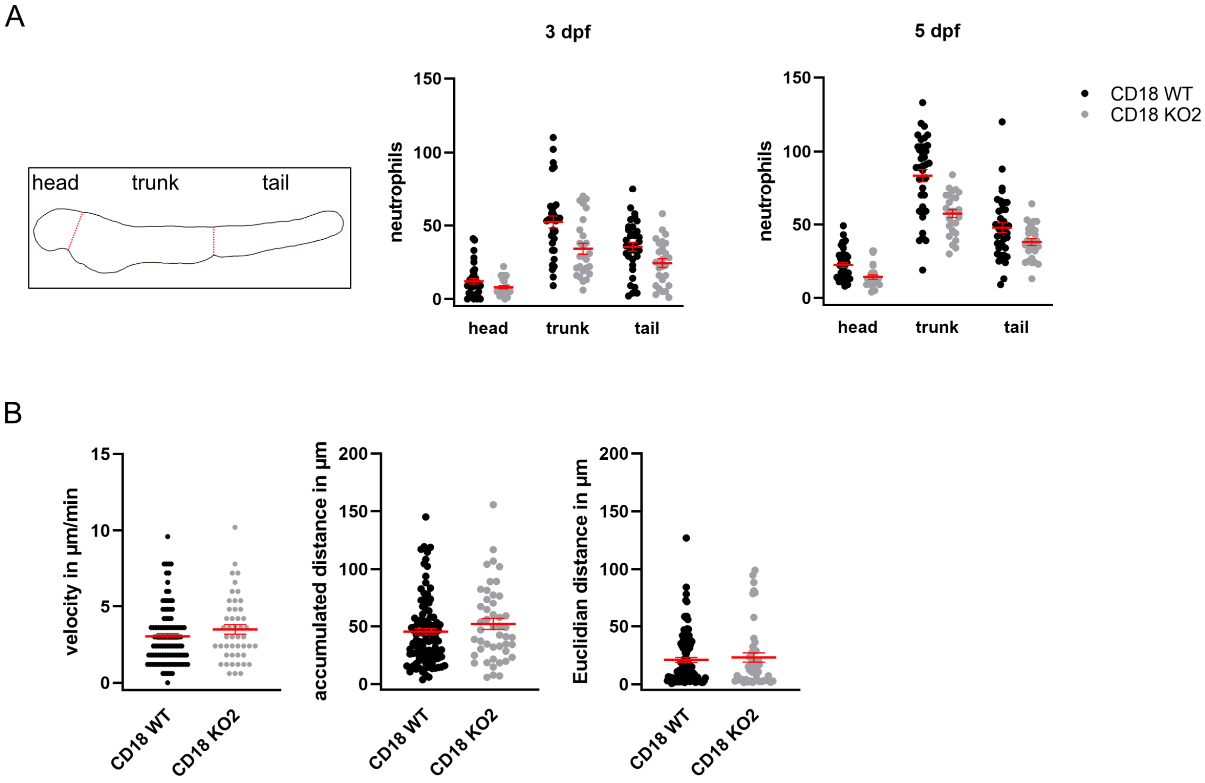


**Supplementary Figure 4: Analysis of neutrophil count and random migration at steady state in CD18 KO2.** (**A**) Left: Schematic of zebrafish larvae for analyzing neutrophil distribution in head, trunk, and tail. Middle and right: Distribution of neutrophils in CD18 WT and CD18 KO2 zebrafish larvae at 3 dpf and 5 dpf. Mean ± sem of ≥ 26 individual larvae of ≥ 3 independent experiments. (**B**) Mean migration velocity, accumulated and Euclidian distance of individual neutrophils. Mean ± sem of ≥ 47 individual neutrophils of 3 independent experiments. Unpaired t-test.

**Supplementary Figure 5**

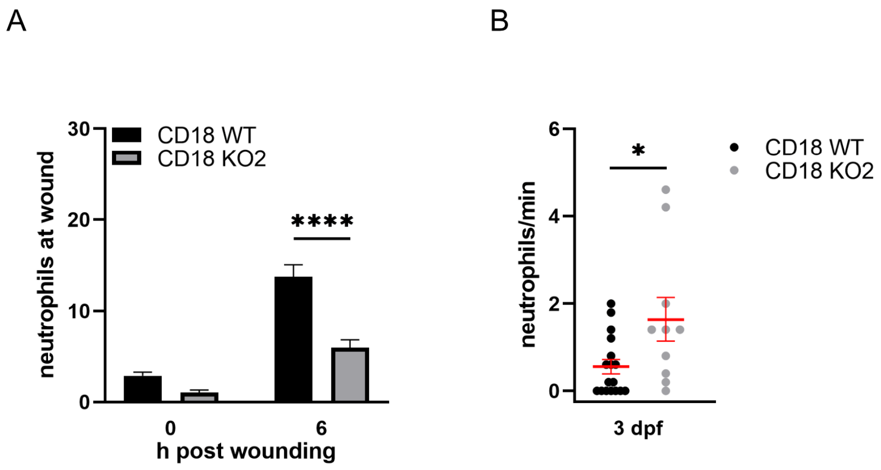


**Supplementary Figure 5: Neutrophil trafficking in CD18 KO2 zebrafish.** (**A**) Quantification of neutrophil numbers at the transected tail fin in 3 dpf CD18 WT and CD18 KO2 zebrafish larvae. Mean ± sem of ≥ 14 individual larvae of ≥ 2 independent experiments. One-way ANOVA, $p < 0.0001$: ****. Similar results were obtained after 5 dpf. (**B**) Number of neutrophils per min in the circulation 6 h after tail fin transection in CD18 WT and CD18 KO2 zebrafish larvae at 3 dpf. Mean ± sem of ≥ 10 individual larvae of 2 independent experiments. Unpaired t-test, $p < 0.05$: *.

**Supplementary Videos**

**Supplementary Video 1: Time-lapse confocal spinning disc maximum z projections showing random migration of neutrophils (red) in zebrafish CD18 WT and CD18 KO1 larvae at 5 dpf.** Endothelial cells are shown in green. Three representative tracks of neutrophils migrating interstitially are shown. Time lapse interval = 60 s. Frame rate = 0.02 frames/s. Scale bar represents 100 µm.

**Supplementary Video 2: Time-lapse confocal spinning disc video microscopy showing neutrophils (red) in the posterior caudal vein 6 h post wounding in CD18 WT and CD18 KO1 zebrafish larvae at 5 dpf.** Endothelial cells are shown in green. Arrows point out neutrophils detected in the circulation. Time lapse interval = 4.5 s. Frame rate = 0.22 frames/s. Scale bar represents 200 µm.
